# Fluorescent riboswitch-controlled biosensors for the genome scale analysis of metabolic pathways

**DOI:** 10.1038/s41598-024-61980-w

**Published:** 2024-05-31

**Authors:** A. Michaud, D. Garneau, J.-P. Côté, D. A. Lafontaine

**Affiliations:** Department of Biology, Faculty of Science, RNA Group, Université de Sherbrooke, Sherbrooke, QC J1K 2R1 Canada

**Keywords:** Riboswitch, Metabolic pathway, Thiamin pyrophosphate, Green fluorescent protein, Bacterial genetics, Non-coding RNAs

## Abstract

Fluorescent detection in cells has been tremendously developed over the years and now benefits from a large array of reporters that can provide sensitive and specific detection in real time. However, the intracellular monitoring of metabolite levels still poses great challenges due to the often complex nature of detected metabolites. Here, we provide a systematic analysis of thiamin pyrophosphate (TPP) metabolism in *Escherichia coli* by using a TPP-sensing riboswitch that controls the expression of the fluorescent *gfp* reporter. By comparing different combinations of reporter fusions and TPP-sensing riboswitches, we determine key elements that are associated with strong TPP-dependent sensing. Furthermore, by using the Keio collection as a proxy for growth conditions differing in TPP levels, we perform a high-throughput screen analysis using high-density solid agar plates. Our study reveals several genes whose deletion leads to increased or decreased TPP levels. The approach developed here could be applicable to other riboswitches and reporter genes, thus representing a framework onto which further development could lead to highly sophisticated detection platforms allowing metabolic screens and identification of orphan riboswitches.

## Introduction

Bacteria need to continually monitor the levels of a large array of structurally and functionally different cellular components to ensure normal growth. Such a diversity of cellular regulators and associated molecular mechanism(s) unavoidably requires regulatory networks to be highly specific so that unwanted crosstalk remains minimal between signaling pathways. In such conditions, cellular metabolites are deeply involved in homeostasis and play various roles such as coenzymes or substrates for enzymatic reactions, and are used for post-translational modifications. Although it still remains challenging, the detection of cellular metabolites is highly desirable and can provide a "metabolic snapshot" about the conditions of bacterial cells^1^. While chromatography-mass spectrometry technologies enable the parallel analysis of cellular metabolites with exquisite precision^1,2^, the use of biosensors allowing the in vivo monitoring of cellular metabolism offers relatively rapid investigation and flexibility in measurements^3^.

Riboswitches are natural ligand sensors that are contained within the 5' untranslated region of messenger RNAs^4–6^. Upon the binding of a target ligand, riboswitches undergo structural changes and modulate gene expression at the levels of transcription, translation or mRNA stability^7^. These RNA sensors are highly specific and often exhibit affinities in the range of cellular concentrations of the detected ligands^8,9^, making them excellent genetic regulators to specifically coordinate the expression of the required gene(s) for cellular homeostasis. Riboswitches are constituted by an aptamer domain that directly binds to the sensed ligand and an expression platform that controls the expression of downstream genes upon ligand binding. Over 55 classes of riboswitches have been discovered to date and represent a diverse range of chemical compounds^4–6^. It is suggested that this ensemble of ligands constitutes a small set of the total number of riboswitch classes^10^, most likely indicating that the diversity of detected ligands should substantially grow in the future. Because riboswitches have evolved to specifically recognize target ligands with high affinity, they represent an attractive aspect for the development of metabolite biosensors. Importantly, riboswitches are readily amenable to reporter gene assays given that separate RNA domains are used for metabolite recognition and genetic regulation.

Recent advances have allowed to study bacterial genetic regulation on a genome scale^11,12^. For instance, a comprehensive library of fluorescent transcriptional reporters has been engineered to contain ~ 2000 *Escherichia coli* promoters and has led to accurately measure dynamic changes of gene expression^12^. Furthermore, a library of ~ 4000 single-gene knockout mutants in *E. coli*, the Keio collection, has permitted the systematic analyses of gene regulatory networks^11^. Clearly, the availability of such resources provides a powerful advantage to study genetic regulation. Furthermore, these genomic libraries offer an unprecedented level of analysis that could be implemented with riboswitch reporter assays to investigate metabolic pathways and associated gene regulation mechanisms. By developing riboswitch reporters, it allows to circumvent limitations of existing methods for analyzing cellular components, such as low dynamic range, low throughput and the need to lyse cells, which can be time consuming^13,14^.

Here, we describe a fluorescent reporter based on the *E. coli thiC* riboswitch and provide a detailed study demonstrating the sensitivity and specificity of TPP sensing^15^. By using such a biosensor, we examine various aspects of regulation such as growth conditions and impact of TPP addition during bacterial growth. Finally, by integrating the Keio collection, we provide an example showing how a TPP biosensor may reveal important aspects of metabolic pathways. This study represents a framework onto which any riboswitch sensors may be used to investigate metabolic pathways on a large scale.

## Results

### Gene regulation mechanisms of the *E. coli thiC* riboswitch

**Figure 1:**
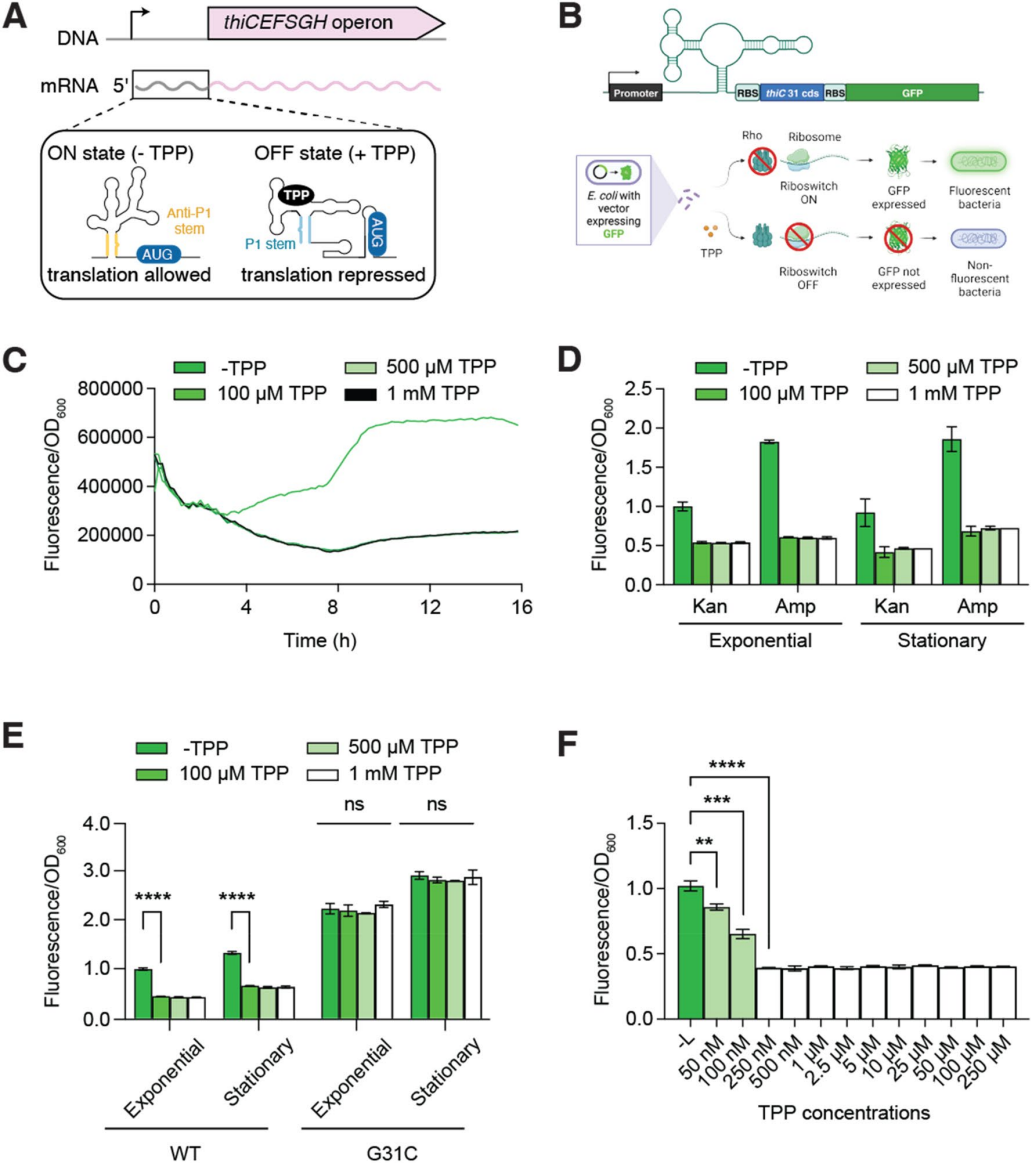
The *Escherichia coli thiC* riboswitch controls *gfp* expression upon TPP sensing. (**A**) Schematic representing the genomic location of the *thiC* riboswitch. The inset shows the ON and OFF riboswitch conformations that are adopted without and with TPP, respectively. While translation initiation is efficient in the absence of TPP (ON state), the riboswitch adopts the OFF state upon TPP binding in which translation initiation is inhibited. The ON and OFF states of the riboswitch correspond to the formation of the anti-P1 and P1 stems, respectively. The AUG start codon is shown. (**B**) Schematic describing the *thiC-gfp* transcriptional fusion. The natural *thiC* promoter is used to express the fusion, which contains the ribosome binding site (RBS) and the 31 first codons of *thiC*. A strong RBS is located immediately upstream of *gfp* to ensure that its expression solely depends on mRNA levels. (**C**, **D**) Expression of the *thiC-gfp* fusion without and with various concentrations of TPP. The expression has been monitored over time in **c** and calculated for the exponential and stationary phases in (**D**). The expression was normalized to the optical density measured at 600 nm. The values determined for the exponential and stationary growth phases were taken at the midpoint of the exponential phase and at 16 h, respectively. The histograms represent values normalized to the expression obtained without ligand. The average values of three independent experiments with SDs are shown. (**E**) Expression of the *thiC-gfp* fusion measured using the WT and the G31C riboswitch mutant. Measurements were performed without and with various concentration of TPP. Values were normalized to the expression obtained without ligand. The average values of three independent experiments with SDs are shown. (**F**) Effect of increasing TPP concentrations on the expression of the *thiC-gfp* fusion during the stationary growth phase (16 h). Values were normalized to the expression obtained without ligand. The average values of three independent experiments with SDs are shown.

The *thiC* TPP-sensing riboswitch in *E. coli* is characterized by an aptamer domain involved in the sensing of TPP (Fig. 1A). When bound to TPP, the aptamer adopts the P1 stem and a stem-loop structure that prevents the initiation of translation by sequestering the ribosome binding site (RBS) and the AUG start codon (Fig. 1A). However, in the absence of TPP, the riboswitch adopts an ON state structure in which the anti-P1 stem is formed, thus allowing the RBS and AUG sequences to be exposed and the efficient initiation of translation^8^. In addition to regulate translation, the *E. coli thiC* riboswitch was previously demonstrated to control transcription elongation through Rho transcription termination^8^. Biochemical probing suggests that the Rho utilization site (*rut*) is located within the aptamer domain at positions U93 to U104 and is more accessible to Rho binding when the riboswitch is bound to TPP^8^. Thus, while the riboswitch modulates translation initiation through the occlusion of the RBS and AUG sequences, the riboswitch also modulates mRNA levels by directly controlling Rho transcription termination. Lastly, it was observed that the promoter activity is not regulated by variations of intracellular TPP^8^, thus making the riboswitch the sole regulator of *thiC* expression.

### The *thiC *riboswitch efficiently detects TPP concentrations in *E. coli*

A library of transcriptional fusions was previously engineered in which ~ 2000 *E. coli* promoters were fused to *gfp* and were expressed from a low-copy plasmid^12^. In that library, the *thiC* promoter, the riboswitch and the first 31 codons of *thiC* were inserted in a transcriptional construct to report on *thiC* mRNA levels (Fig. 1B, upper panel). The plasmid contains a kanamycin resistance gene and a fast-folding *gfpmut2* gene with a strong RBS^12^. Using this construct, it is expected that in the absence of TPP, Rho transcription termination is not activated^8^ and that translation initiation is efficient, thus leading to high GFP fluorescence (Fig. 1B, lower panel). However, the presence of TPP in the medium should allow the TPP-bound riboswitch to adopt a conformation leading to Rho transcription termination and the inhibition of translation, ultimately preventing GFP fluorescence (Fig. 1B, lower panel). Given that Rho transcription termination is involved in the riboswitch regulation mechanism, we reasoned that using such a transcriptional reporter should allow us to monitor TPP levels.

To test this transcriptional fusion, we first incubated cells in a minimal medium with various concentrations of TPP and measured the expression of the *gfp* fusion over a time course of 16 h. By simultaneously monitoring the OD_600_ and the GFP fluorescence, we obtained the GFP fluorescence emission normalized to the cellular growth (fluorescence/OD ratio). In the absence of supplemented TPP, we found that the expression of *gfp* gradually increased during the exponential growth phase and was stabilized at 8 h, corresponding to the onset of the stationary phase (Fig. 1C and see constructs in Supplementary Fig. 1). When repeating these experiments in the presence of 1 mM TPP, we found that the expression of *gfp* was decreased by at least ~ twofold both in the exponential and stationary growth phases (Fig. 1C,D). Similar decreases in *gfp* activity were observed when using lower concentrations (100 and 500 µM), suggesting that TPP is at saturating levels in these conditions. These experiments are in agreement with previous studies showing that TPP decreases the expression of *thiC* in *E. coli*, consistent with the riboswitch regulatory mechanism^8,16,17^.

In order to use the *gfp* transcriptional construct in the Keio collection^11^, the kanamycin resistance gene, present in the Keio mutants, was replaced with a gene conferring resistance to ampicillin (Supplementary Fig. 2). The ampicillin resistance gene introduced in the plasmid ensured a dual antibiotic selection in the context of the Keio mutants, thereby to select for both the presence of the plasmid and the Keio mutants. When monitoring the expression of *gfp* with the new construct, we obtained very similar results with and without TPP added as a supplement in the medium both in exponential and stationary growth phases (Fig. 1D), suggesting that changing the resistance gene does not perturb TPP detection by the riboswitch. Thus, the ampicillin-resistant construct was used for further study.

To establish whether the decrease in GFP fluorescence was due to the riboswitch activity, we engineered an additional transcriptional construct in which TPP binding was disabled by the introduction of the G31C mutation^8^. According to crystal structures from the similar *thiM* riboswitch^18,19^ and the *thiC* riboswitch from *Arabidopsis thaliana*^20^, the G31 residue is located in the ligand binding site and directly contacts the bound TPP. Consequently, the G31C mutation is quite detrimental for the riboswitch regulation mechanism^8,21^. When using the G31C mutant, the expression of *gfp* was not diminished in the presence of TPP (Fig. 1E), consistent with this mutation preventing TPP sensing. In the absence of supplemented TPP, we found that the expression was increased by ~ 2.2-fold compared to the WT construct (Fig. 1E). These results suggest that, in the context of the WT construct, a relatively low TPP concentration is detected by the reporter construct thereby resulting in a lower *gfp* expression than in the G31C mutant. Alternatively, these data could also imply that the G31C mutation induces the riboswitch to adopt to a greater extent the ON state structure, which would result in gene expression being favored.

To establish the sensitivity of the *thiC-gfp* transcriptional construct, we tested the effect of TPP concentrations lower than 100 µM, which was shown to efficiently decrease *gfp* expression (Fig. 1E). When cells were grown in minimal medium supplemented with TPP concentrations ranging from 50 nM to 250 µM, we observed that riboswitch sensing was detected with the lowest concentrations used in the stationary growth phase (Fig. 1F). For instance, although a small regulation was detected at 50 nM, a ~ 1.5-fold decrease in regulation was obtained when supplementing 100 nM TPP. The efficiency of regulation was maximally when supplemeting 250 nM (Fig. 1F), which represents a ~ tenfold increase in sensitivity compared to previous in vitro transcription-translation-coupled assays^8^. These results are in good agreement with the in vivo TPP concentration being in the low micromolar range^22^, thus making the riboswitch well-tuned to detect cellular TPP variations in *E. coli*.

### Microscopy analysis of TPP sensing

**Figure 2:**
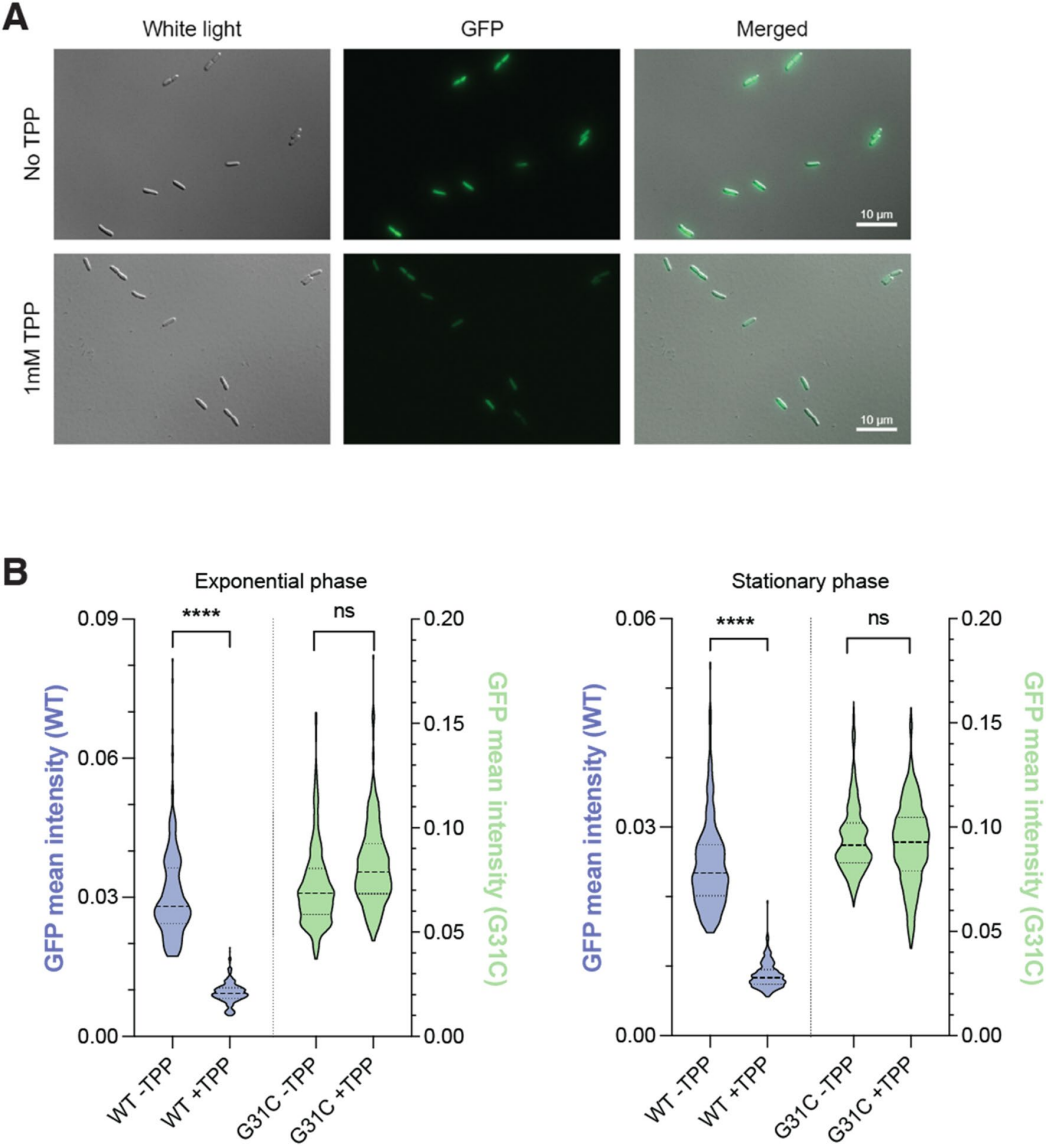
Microscopy analysis of riboswitch TPP sensing. (**A**) Microscopy images obtained using brightfield and GFP fluorescence. Merged images are obtained by using manual alignment of brightfield and fluorescence images. Experiments were performed without and with supplemented 1 mM TPP. (**B**) Violin plots of the microscopy analysis obtained without and with supplemented 1 mM TPP at the exponential (left) and stationary (right) phases. The WT (blue) and G31C (green) transcriptional fusions were used to obtain the data. The GFP intensity of the WT and G31C are indicated on the left and right of each graph, respectively. The middle line indicates the median of the data. The upper and lower divisions correspond to the first and third quartiles, respectively.

Following the liquid assays, a microscopy confirmation was performed to validate the results (Fig. 2A). This allowed us to confirm the expression of GFP and the distribution of the bacterial expression at the cellular level. The presence of TPP reduced the fluorescence expression by ~ 69% in exponential phase and ~ 65% in stationary phase for the WT construct (Fig. 2B), consistent with the results obtained in liquid culture (Fig. 1E). As expected, the G31C binding mutant did not allow TPP regulation (Fig. 2B). These data also showed that the fluorescence expression or the presence of TPP did not affect the cellular growth nor their size or shape (data not shown). The GFP is expressed throughout the cell and is not localized in a particular region. It can be observed that while not all bacteria expressed the GFP at the same level, the results show quite a homogenous population as shown on Fig. 2B.

### Effect of TPP measurements on the efficiency of regulation

**Figure 3:**
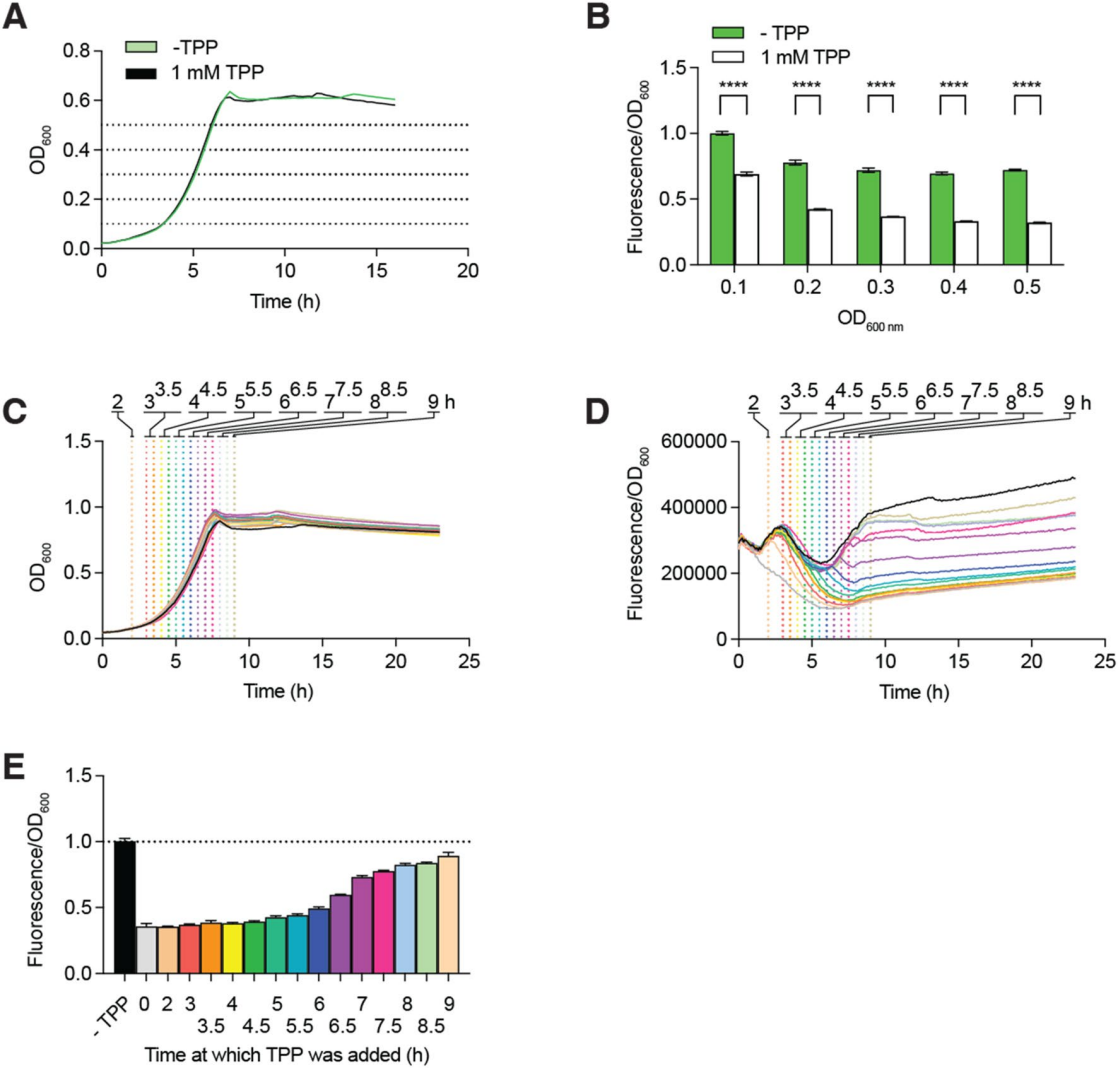
Characterization of the TPP-induced riboswitch regulation. (**A**) Growth curves of *E. coli* cells without and with supplemented 1 mM TPP. The growth has been monitored by measuring the optical density at 600 nm. (**B**) Fluorescence intensity of the *thiC-gfp* fusion calculated at the indicated optical density. Values were normalized to the expression obtained without ligand at OD_600_ of 0.1. The average values of three independent experiments with SDs are shown. (**C**, **D**, **E**) Growth curves of *E. coli* cells and expression of the *thiC-gfp* fusion without and with supplemented 1 mM TPP. The cellular growth has been monitored by measuring the optical density at 600 nm in (**C**). The expression of the fusion has been normalized to the optical density in (**D**) and has been calculated for the stationary phase in (**E**). The histograms represent values normalized to the expression obtained without ligand. The average values of three independent experiments with SDs are shown. The addition of TPP has been performed at the indicated time during the growth.

Our fluorescence assays suggest that the effect of TPP on riboswitch regulation is similar when measurements are performed either in the exponential or stationary phase (Fig. 1E). To establish the kinetics of TPP-dependent riboswitch regulation, we measured the expression of *gfp* without and with supplementation of 1 mM TPP at earlier time points during the exponential phase. While measurements performed at an OD of ~ 0.1 revealed a lesser efficiency of riboswitch regulation (~ 1.5-fold), data obtained at an OD of ~ 0.2 and higher showed a very similar effect, ranging from ~ 1.9 to ~ 2.2-fold (Fig. 3A,B). These results indicate that most of the TPP regulation is obtained early during the exponential growth phase.

In our assays, the effect of TPP on *gfp* expression is monitored by adding the metabolite early in the exponential phase (OD ~ 0.1). To determine the importance of TPP being detected by the riboswitch early during cellular growth, we evaluated the effect of adding TPP at various time points during the growth phase. For these experiments, the efficiency of regulation was measured in the stationary phase. No effect on growth was observed when TPP was added at the tested time points (Fig. 3C). When TPP was added during the first half of the exponential phase (from 0 to 6 h), the expression of *gfp* was repressed at least by a factor of ~ twofold (Fig. 3D,E). However, when adding the metabolite at later time points, the regulation was found to gradually diminish and to attain a plateau at ~ 8 h where regulation was almost negligible (Fig. 3D,E). The lack of riboswitch regulation observed when TPP was added at late time points suggests that TPP must be sensed relatively early during the growth phase for the regulation of *gfp* expression to be efficient. For the rest of the study, riboswitch regulation was assayed by adding TPP at an OD of ~ 0.1 and by measuring *gfp* expression during exponential and stationary phases.

### Modularity of the TPP riboswitch sensor

**Figure 4:**
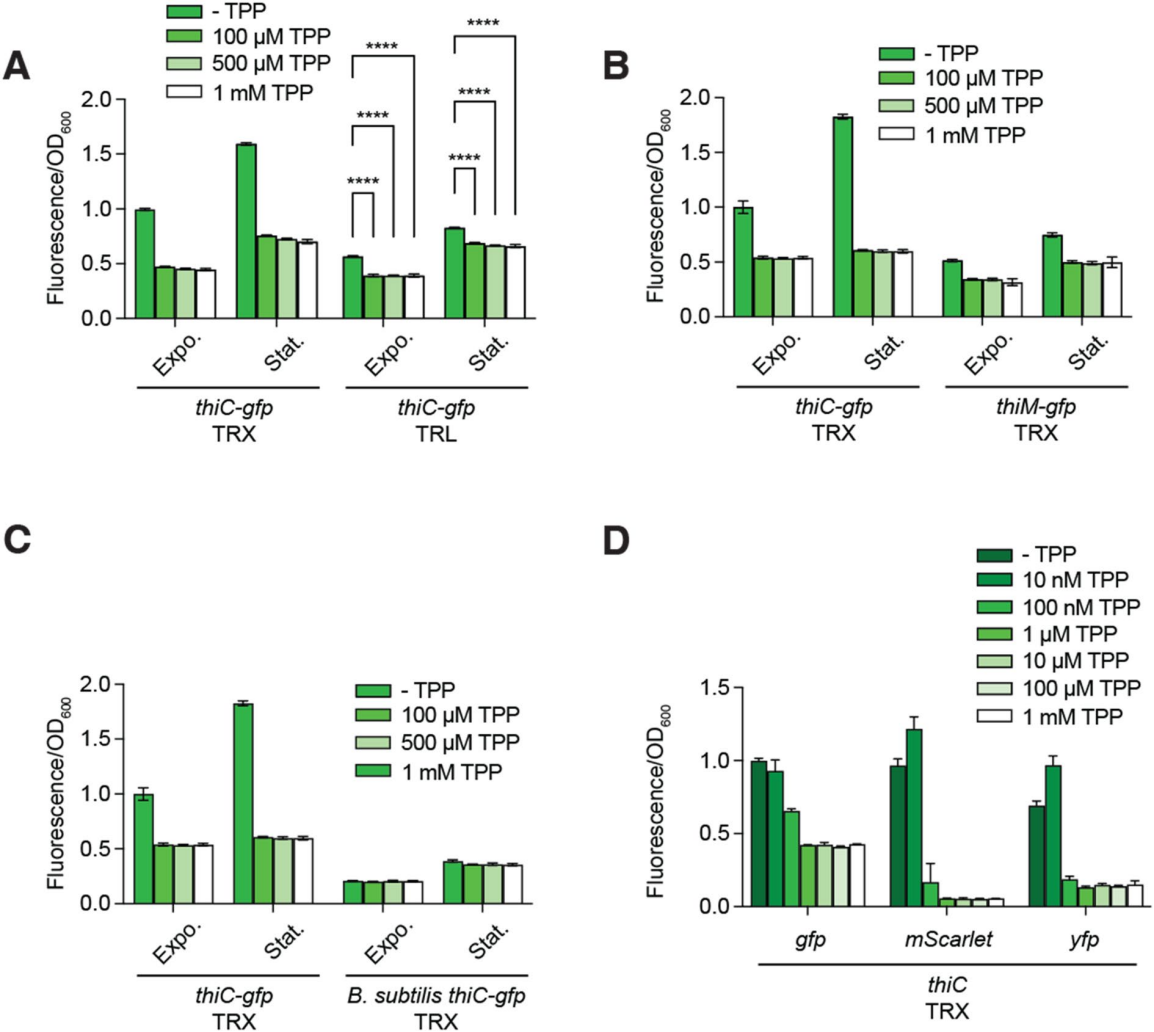
Comparison of various riboswitches and reporter genes for TPP sensing. (**A**) Expression of the *E. coli thiC-gfp* transcriptional (TRX) and ThiC-GFP translational (TRL) fusions without and with various supplemented TPP concentrations. Values were normalized to the expression obtained without ligand. The average values of three independent experiments with SDs are shown. (**B**) Expression of the *E. coli thiC-gfp* and *thiM-gfp* transcriptional fusions without and with various supplemented TPP concentrations. Values were normalized to the expression obtained without ligand. The average values of three independent experiments with SDs are shown. (**C**) Expression of the *E. coli thiC-gfp* and *B. subtilis thiC-gfp* transcriptional fusions without and with various supplemented TPP concentrations. Values were normalized to the expression obtained without ligand. The average values of three independent experiments with SDs are shown. (**D**) Expression of the *E. coli thiC-gfp*, *E. coli thiC-mScarlet* and *E. coli thiC-yfp* transcriptional fusions without and with various supplemented TPP concentrations. Values were normalized to the expression obtained without ligand. The average values of three independent experiments with SDs are shown.

Because the architecture of riboswitches is modular, several variations of the TPP riboswitch exist in which either the aptamer or the expression platform differ in sequence and structure. To determine whether TPP sensing was optimally performed in the context of a *gfp* transcriptional fusion controlled by the *E. coli thiC* riboswitch, we assessed several other configurations to report on TPP detection. We first evaluated the use of a translational fusion in which *gfp* translation initiation is directly controlled by the *thiC* riboswitch expression platform. In the translational construct, the RBS of *gfp* is effectively replaced with the natural RBS sequence of *thiC*. When growing cells in absence of supplemented TPP, the expression of the construct was found to be lower by a factor of ~ 1.8-fold compared to the transcriptional construct (Fig. 4A, see both exponential and stationary phases). These results indicate that the expression of *gfp* in the translational construct is less efficient than in the transcriptional construct. This increased gene expression in the transcriptional fusion is most probably due to the use of a strong RBS^12^. Importantly, when cells were grown in the presence of supplemented TPP, small decreases in *gfp* expression were detected both in the exponential (~ 1.4-fold) and stationary (~ 1.2-fold) phases (Fig. 4A), showing that the expression is slightly reduced when adding TPP. These results show that the *thiC* translational construct exhibits a smaller TPP-induced regulation, thereby representing a less suitable biosensor for cellular detection.

Next, we used the *thiM* riboswitch of *E. coli* that has been reported to sense TPP^15,21,23^. The secondary structure of the *thiM* aptamer domain is very similar to that of *thiC* and has been reported to have a dissociation constant lower than the *thiC* variant^15^. To compare to the *E. coli thiC-gfp* transcriptional fusion, the *thiM* riboswitch and the first 31 codons of *thiM* were used to replace the corresponding *thiC* region in the reporter construct. When monitoring the expression of the *thiM-gfp* construct, we found that similarly to the *thiC* translational construct (Fig. 4B), the expression of the *thiM-gfp* fusion was decreased by ~ 2.0-fold and ~ 2.4-fold in the exponential and the stationary phase, respectively, when compared to the *thiC-gfp* construct (Fig. 4B). When the expression of the *thiM-gfp* fusion was measured in the presence of TPP, we observed that it decreased by ~ 1.6-fold and ~ 1.5-fold in the exponential and stationary phases, respectively (Fig. 4B). These data indicate that the TPP effect obtained with the *thiM-gfp* transcriptional construct is not optimal compared to the *thiC-gfp* transcriptional fusion.

Given that a better regulation was monitored when using the transcriptional version of the *thiC-gfp* fusion (Fig. 4A), we reasoned that a transcriptionally-regulating riboswitch could lead to a stronger repression than the *E. coli thiC* riboswitch, which primarily regulates at the translational level (Fig. 1A)^8^. A new construct was designed to monitor the regulatory activity of the *thiC* riboswitch from *Bacillus subtilis*, which performs transcriptional regulation by modulating an intrinsic terminator^15^. The new construct contained the corresponding *thiC* riboswitch and the first 31 *thiC* codons from *B. subtilis*. When monitoring this construct in the absence of supplemented TPP, the expression of *gfp* was found to be reduced by ~ 2.0-fold and ~ 2.5-fold in the exponential and stationary phases when compared to the *E. coli thiC* riboswitch (Fig. 4C). Furthermore, when the experiments were repeated in the presence of TPP, no decrease in *gfp* expression was detected (Fig. 4C), indicating that the construct is not sensitive to TPP. These results suggest that the *B. subtilis* riboswitch is not suitable to monitor TPP levels in *E. coli*.

### Importance of the fluorescent protein in metabolite detection

Given the availability of several fluorescent proteins, we tested whether TPP sensing could be improved by using other fluorescent proteins. To do so, we used the fluorescent reporters mScarlet^24^ and YFP^25^ that exhibit higher brightness properties. When replacing the *gfp* region with the *mScarlet* reporter, thus representing a *thiC-mScarlet* transcriptional fusion, we found that the TPP-induced expression was strongly reduced by a factor of ~ 19-fold in the stationary phase (Fig. 4D). These results show that the use of mScarlet increases the detection dynamic range by ~ 7.5-fold when compared to *gfp* (Fig. 4D), thus suggesting that *mScarlet* represents a better reporter than *gfp* in *E. coli*. Similarly, when using a transcriptional construct containing the *yfp* reporter, the regulation efficiency obtained in the presence of TPP (~ 4.7-fold) was more important than when using *gfp* (Fig. 4D). Together, these results indicate that the use of both *mScarlet* and *yfp* reporters allow to obtain a larger dynamic range when compared to *gfp*.

### Exploring the TPP metabolism using a riboswitch biosensor

**Figure 5:**
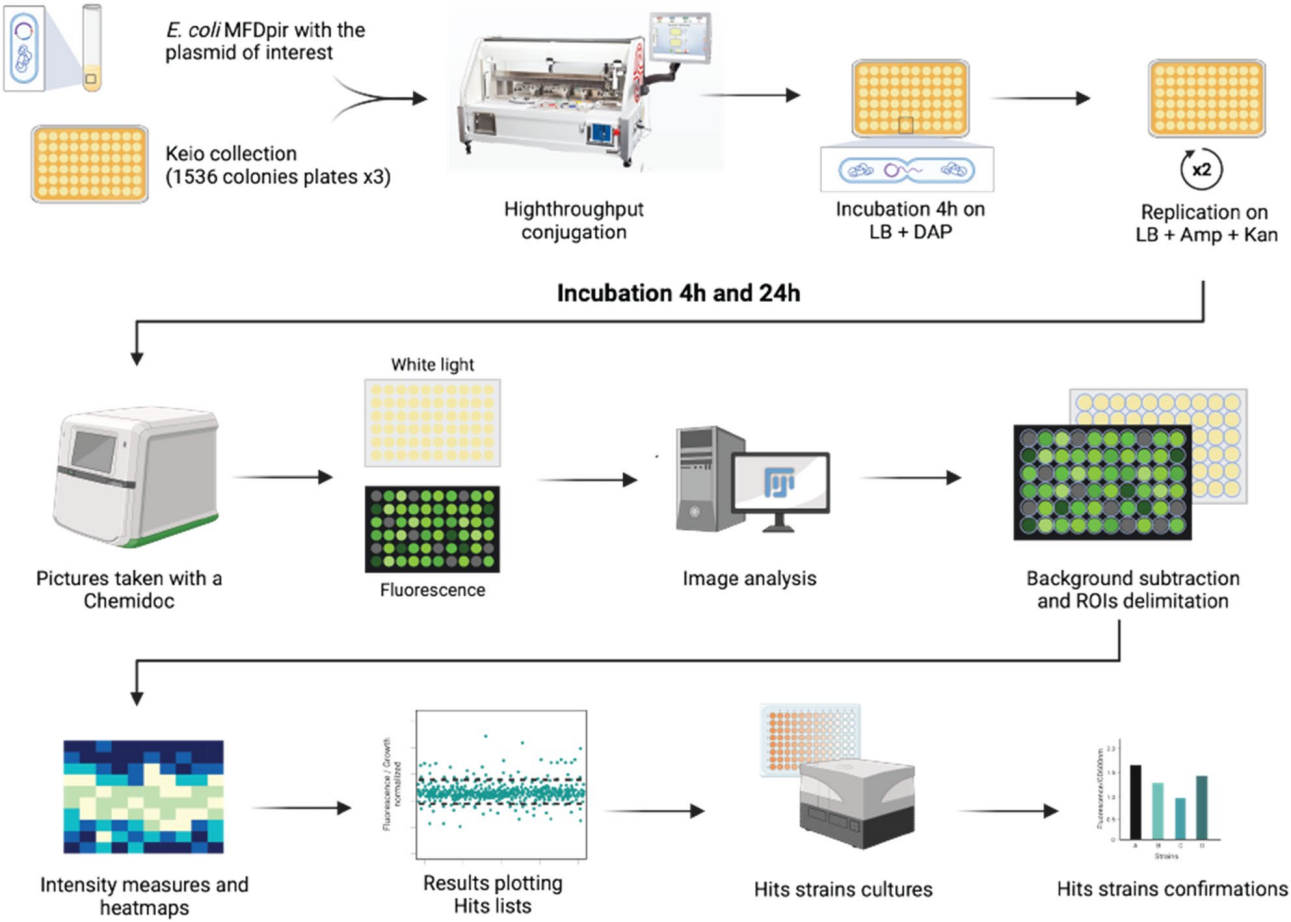
Overall approach for the high-throughput screen analysis of TPP levels in the Keio collection. The overall approach starts with high-throughput conjugation of the Keio collection with the plasmid containing the *thiC-gfp* transcriptional fusion using a robot Rotor HDA. An incubation is performed to allow the conjugation to be efficient, which is then followed by a selection process to retain the plasmid in the strains, while allowing the removal of the MFD*pir* strain. Pictures of the resulting fluorescent strains are taken at 4 and 24 h using white and fluorescent lights. Images are analyzed using the Fiji software where the background of the fluorescence is removed and the colonies are delimited. Their intensity is extracted and is used to produce heatmaps and index of expressions. The hits are then identified and confirmed using liquid assays.

To investigate the metabolism of TPP in *E. coli*, we performed a high-throughput conjugation of the plasmid-containing *thiC-gfp* transcriptional construct with the Keio collection in 1536-density plates^26,27^. This transcriptional fusion was selected due to the high extent of TPP-induced regulation that it produced compared to the translational construct and to the other riboswitches (Fig. 4A,B). Furthermore, the *thiC-gfp* construct was preferred over the *mScarlet* fusion that yielded stronger TPP-dependent downregulation (Fig. 4D) given that our detection system for high-throughput experiments can measure GFP but not the other fluorophores. Briefly, the plasmid-containing strain was grown in LB with ampicillin and pinned on agar plates containing the Keio collection (Fig. 5). Plates were incubated for 1 h before being pinned onto ampicillin/kanamycin-supplemented LB agar to select the Keio strains (Kan^R^) expressing the *thiC-gfp* fusion construct plasmid (Amp^R^). The newly conjugated strains were then pinned on fresh agar plates and incubated at 37 °C for 24 h, and images were collected at 4 h and 24 h of growth (Supplementary Fig. 3A). An image analysis script was used to quantify the colony biomass at each time point and was compiled into growth curves. Pre- and post-normalization of the plates and associated density histograms showed that the spatial edge and incubator effects (border effect) were efficiently removed (Supplementary Fig. 3B,C)^26^. In addition to the growth, the fluorescence of each strain was monitored during the assays and the obtained images were analyzed using the Fiji software^27^.

Using the recorded fluorescence and colony biomass, the fluorescence/growth ratio was calculated for each mutant strain of the Keio collection and the obtained data were combined to recreate a heatmap representing the plate configuration, in which empty wells were removed. It can be observed that the range of *gfp* expression is uniformly distributed and not concentrated at the plate border, consistent with the efficiency of the normalization algorithm (Supplementary Fig. 4A)^26^. A Pearson analysis revealed that the data was reproducibly obtained across three biological replicates (Supplementary Table 1). Furthermore, by taking advantage that 402 mutant strains were duplicated on the agar plates, we compared the *gfp* expression of corresponding duplicate strains as an internal control (Supplementary Fig. 4B). The majority of duplicates have very similar levels of *gfp* expression that are similar between the duplicates. Thus, these data suggest that the approach to monitor the *gfp* expression on high-density agar plates is robust and reproducibly detect gene expression in similar experimental conditions.

**Figure 6:**
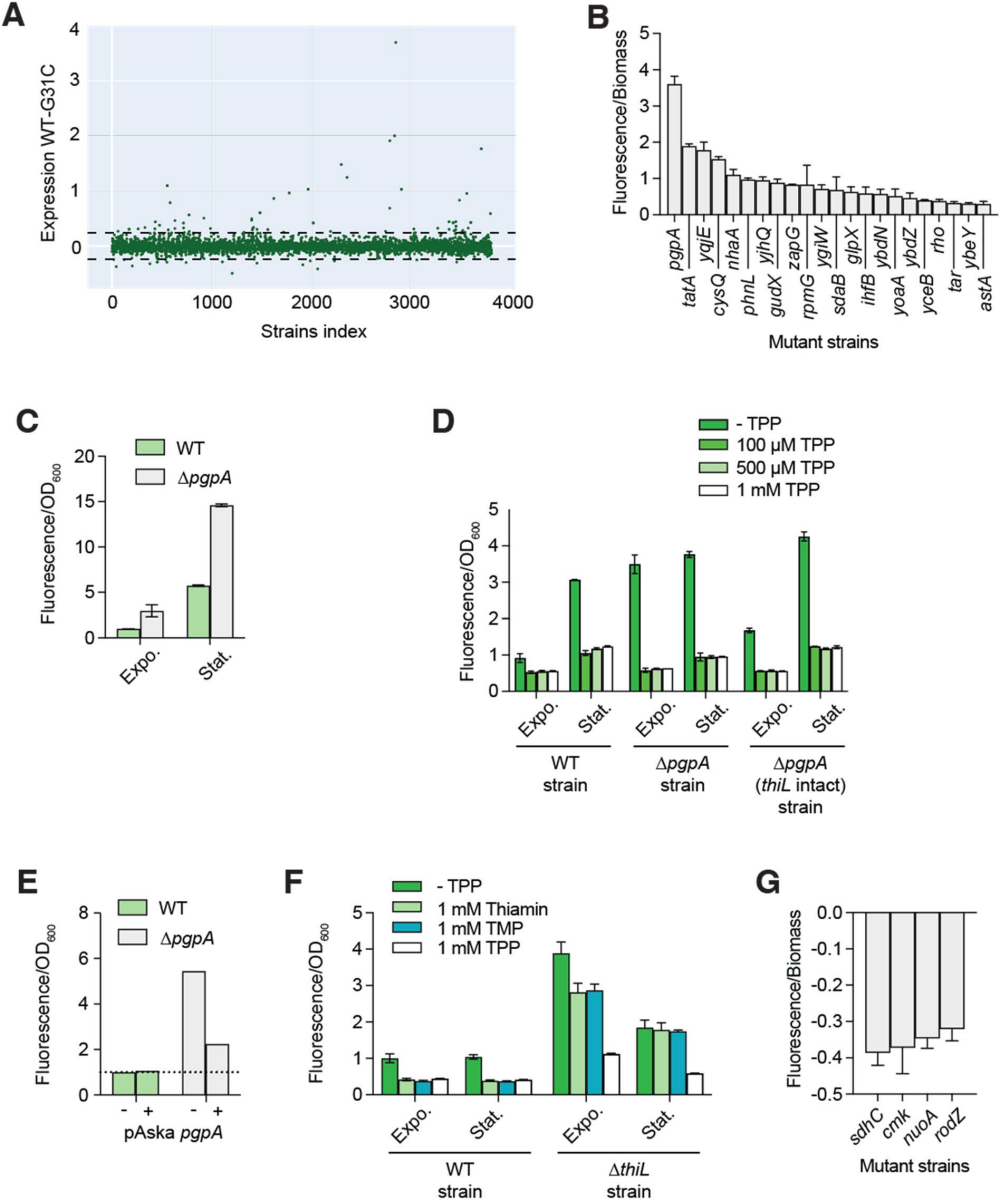
High-throughput analysis of TPP levels in the Keio collection. (**A**) Index plot showing the ratio *gfp* expression/strain biomass for the Keio collection. Screens were performed using the WT *thiC-gfp* transcriptional fusion and the G31C mutant of the fusion to remove any effect unrelated to riboswitch regulation. Data showing expression values higher or lower than 5 standard deviations (dotted lines) were considered significantly different than the average. (**B**) Hits list representing the 22 values exhibiting a *gfp* expression that is at least higher than 5 standard deviation errors according to the high-throughput screen. The average values of three independent experiments with SDs are shown. (**C**) Expression of the *E. coli thiC-gfp* transcriptional in the WT and *∆pgpA* mutant strain in LB. Values were normalized to the expression obtained without ligand. The average values of three independent experiments with SDs are shown. (**D**) Expression of the *E. coli thiC-gfp* transcriptional in the WT, *∆pgpA* and *∆pgpA* (intact *thiL*) mutant strains in minimal medium. Expressions were measured without and with various supplemented TPP concentrations. Values were normalized to the expression obtained without ligand. The average values of three independent experiments with SDs are shown. (**E**) Expression of the *E. coli thiC-gfp* transcriptional in the WT and *∆pgpA* mutant strain in minimal medium. Experiments were performed without and with the expression of *pgpA* from a plasmid. Values were normalized to the expression obtained without ligand. The average values of three independent experiments with SDs are shown. (**F**) Expression of the *E. coli thiC-gfp* transcriptional in the WT and *∆thiL* mutant strain in the presence of 1 mM thiamin, thiamine phosphate and TPP. Values were normalized to the expression obtained without ligand. The average values of three independent experiments with SDs are shown. (**G**) Hits list representing the 4 values exhibiting a *gfp* expression that is at least lower than 5 standard deviation errors according to the high-throughput screen. The average values of three independent experiments with SDs are shown.

We next performed an identical screen but in which a G31C inactive riboswitch fusion was used to filter out the effects that are not directly related to riboswitch sensing (Supplementary Fig. 5A,B). By combining the screens obtained from the WT and G31 *thiC-gfp* fusions, a gene expression index was created depicting the TPP levels across the Keio collection through riboswitch-driven fluorescence detection (Fig. 6A). The normalized *gfp* fluorescence acquired from LB agar plates revealed that most of the strains exhibited a fluorescence signal that was within 5 standard error deviations from the average (Fig. 6A). These results suggest that the intracellular TPP levels are not modulated to a high degree in the majority of the Keio collection mutants.

However, in some cases, the gene expression index revealed that the TPP levels were either above or below the threshold after a 24 h growth period. When analyzing the data obtained from three independent biological replicates, a list of hits was generated that correspond to mutant strains showing a high *gfp* fluorescence (Fig. 6B). Importantly, since the *thiC-gfp* TPP biosensor is a repressor switch (Fig. 1A), it therefore suggests that the detected high fluorescence corresponds to low TPP levels in the corresponding mutant strains. The strongest hit observed consists in the *∆pgpA* strain, which exhibits ~ twofold more *gfp* expression than the second most important hit (Fig. 6B). The enzyme *pgpA* encodes phosphatidylglycerophosphatase A that catalyzes the dephosphorylation of phosphatidylglycerol phosphate to phosphatidylglycerol, an essential phospholipid of the inner and outer *E. coli* membrane^28^. Interestingly, *pgpA* is located in the distal part of a four-gene operon where it partially overlaps with *thiL* (Supplementary Fig. 6), which is involved in the conversion of thiamin monophosphate (TMP) to TPP^29^. Control experiments showed that the *∆pgpA* strain also exhibits high fluorescence when grown in LB medium (Fig. 6C), consistent with the data obtained on agar plates (Fig. 6B).

Because the *∆pgpA* mutation deletes 20 nt from the 3' extremity of *thiL*, we were concerned that it might disturb *thiL* expression and we thus engineered a new *∆pgpA* strain that left the *thiL* locus intact (see “Methods”). When monitoring *gfp* expression for both *∆pgpA* strains in a minimal medium without added TPP, the expression was increased for both strains when compared to the WT (Fig. 6D). Thus, these results are in agreement both with the high *gfp* expression observed for the *∆pgpA* strain in the high-density screen data (Fig. 6B) and the increased expression mainly occurring from *pgpA* deletion. Furthermore, when monitoring the *gfp* expression in the presence of TPP, we found that the expression was reduced for the original and reconstructed *∆pgpA* strains (Fig. 6D), indicating that TPP regulation exhibit similar trends in both strains. Lastly, the increased *gfp* expression observed in the *∆pgpA* strain was reduced when expressing *pgpA* from the pCA-24n-*pgpA* plasmid obtained from the Aska collection^30^ (Fig. 6E), suggesting that the lower TPP levels observed in *∆pgpA* can be rescued by expressing *pgpA*. Together, these results suggest that the deletion of *pgpA* results in lower TPP levels, which is reported by the riboswitch construct.

Given that the high-throughput screen conducted in LB agar did not reveal the ∆*thiL* mutant as a strain showing high TPP levels, we next investigated whether TPP levels would be affected in minimal growth conditions. To do so, we introduced the *thiC-gfp* transcriptional fusion in the *∆thiL* strain and monitored the effect of thiamin, TMP and TPP on *thiC-gfp* expression in a minimal medium. While efficient regulation was observed in the WT strain when using the three thiamin compounds (Fig. 6F), TPP was the only metabolite reducing the expression of the fusion in the *∆thiL* mutant strain (Fig. 6F). These results are consistent with TMP not being converted to TPP in the *∆thiL* strain, thus indicating that the TPP-sensing riboswitch biosensor successfully monitors intracellular levels of TPP.

### Strains showing variations in TPP intracellular levels

In addition to *∆pgpA*, the high-throughput screen revealed several mutant strains that showed a high fluorescence signal (low TPP levels) (Fig. 6B). For instance, after *∆pgpA*, the second most important candidate was *∆tatA* that is part of a twin arginine protein translocation system and that may form a complex with *thiB*, which is involved in thiamin uptake^31^. Although less is known about the third most important hit, *yqjE* is an inner membrane protein with two predicted transmembrane domains that has been associated to persistence and the alarmone ppGpp^32^, suggesting that it may be involved in TPP extracellular detection. Other hits potentially related to thiamin import were also identified, such as *nhaA* that is an antiporter mediating the uptake of protons^33^ and *phnL* that is part of a 14-gene operon involved in phosphate uptake and metabolism^34^. The screen also identified *sdaB*, a serine deaminase producing pyruvate, which is a substrate used for thiazole^35^.

A relatively small number of strains exhibiting lower expression of the *thiC-gfp* fusion, thus high TPP levels, were identified in the high-throughput screen (Fig. 6G). The first hit corresponds to *sdhC* that encodes a succinate dehydrogenase that is involved in the TCA cycle^36^. Interestingly, mutations of the corresponding *sdh* gene in *S. typhimurium* alters thiamin requirements for growth^37^. The second hit found was *cmk*, which is a cytidylate kinase involved in pyrimidine ribonucleotide^38^. The last two hits are related to the cellular membrane: *nuoA* that is part of the inner membrane component of NADH dehydrogenase I^39^ and *rodZ*, which is a bitopic inner membrane protein involved in the maintenance of cell shape through interaction with the MreB cytoskeleton^40^. More work will be required to determine the molecular mechanisms involved in maintaining TPP cellular homeostasis.

### TPP does not impact small regulatory RNA mutant strains

We took advantage of the fact that our Keio collection also contained the Storz collection^41^, a collection of small regulatory RNA and small proteins mutant strains, to test whether those had an impact on TPP levels. The 125 mutants are performed into *E. coli* MG1655 to study cellular phenotypes associated with cell wall stresses. Our results suggest that the mutations do not have a significant effect on the TPP concentration. Thus, while functions of sRNAs remain vastly unknown, no functional link to the TPP metabolism was found using our assays (Supplementary Fig. 7).

## Discussion

In this study, we have characterized a riboswitch biosensor reporting on the levels of intracellular TPP. By characterizing various aspects of the riboswitch regulation, we have determined that the use of the *E. coli thiC* riboswitch was the best to detect TPP variations in the nM range in *E. coli* (Fig. 1F). Importantly, while it may be intuitive to use a translational fusion to control *gfp* expression—given that it elicits both transcriptional and translational regulatory mechanisms—our work has determined that a transcriptional construct is best to report TPP levels in our assays (Fig. 4A). Specifically, our data revealed that the strong RBS used in the transcriptional fusion^12^ ensures robust *gfp* expression in the absence of supplemented TPP compared to the natural *thiC* RBS of the translational fusion (Fig. 4A), thus presumably allowing to monitor a greater dynamic regulation range. A reduced expression was also observed in the absence of supplemented TPP for the *E. coli thiM* transcriptional fusion (Fig. 4B), making it less efficient for monitoring TPP levels. This is reminiscent of the natural variability in the regulation efficiency observed among *B. subtilis S*-adenosylmethionine riboswitches^9^. Furthermore, the construct harboring the *B. subtilis thiC* riboswitch resulted in a very low expression regardless of TPP (Fig. 4C), suggesting that the mRNA is either unstable or that the riboswitch is folding in a conformation preventing *gfp* expression. The latter case could be explained by the fact that *B. subtilis* and *E. coli* RNA polymerase do not always respond to the same transcriptional pause signals, which could make an inactive *B. subtilis thiC* riboswitch when transcribed in *E. coli*^42^. Our data have also shown that the use of *mScarlet* is allowing to significantly reduce the fluorescence signal in TPP-saturating conditions (Fig. 4D). One potential cause for the higher fluorescence "background" that is obtained with GFP might be that cells produce an autofluorescence that perturbs the detection of GFP fluorescence^43^. Together, the large differences of TPP-induced regulation observed among the different constructs suggest that care must be taken when attempting to design a biosensor.

Although our fluorescence assays have revealed that TPP detection is efficiently achieved in the nM range (Fig. 1F), it should be possible to increase the sensitivity of the reporter to detect even lower TPP concentrations. As previously reported for *thiC* and other riboswitches^8,44–47^, metabolite recognition is primarily achieved at the cotranscriptional level and often occurs during a small transcriptional window. Such a kinetic regime for metabolite sensing intrinsically results in the speed of transcription being crucial for sensing where slower RNAP elongation rates should allow more time for metabolite binding. Given that the *E. coli thiC* riboswitch exhibits multiple pause sites within the expression platform^8,17,48^, it should be possible to engineer constructs containing stronger pause sites^49^ within the riboswitch. Such constructs should be more efficient to perform cotranscriptional sensing by increasing the available time for TPP to bind and should therefore theoretically exhibit increased efficiencies in detecting lower TPP concentrations.

The high-throughput screen analysis performed on agar plates suggests that the *thiC-gfp* fluorescent biosensor is able to detect TPP variations in *E. coli*. Importantly, even though the Keio collection provides a large set of mutant strains, it is expected that only a small subset of strains should exhibit perturbations of the TPP metabolism. Furthermore, since the collection does not contain any mutant deleted for essential genes, it further restricts the number of candidates that could give useful information about the TPP metabolism. Nevertheless, the screen successfully identified several genes involved in TPP transport and biosynthesis and provided potential insights into interesting molecular mechanisms involving TPP and the TCA cycle, which contains several TPP-dependent enzymes. The use of the G31C riboswitch mutant is particularly important to remove any undesired effect affecting the fluorescence that is not related to the TPP metabolism. For instance, a very low fluorescence signal was observed for the *∆dnaT* and *∆priA* mutant strains when using both the WT and G31C riboswitch fusions, indicating that these mutants perturb GFP fluorescence. Indeed, both *dnaT* and *priA* are involved in DNA replication^50^, suggesting that the replication of the reporter plasmid might be less efficient in these mutant strains.

The approach developed here can in principle be applied using any of the > 55 riboswitch classes^4–6^, providing that the corresponding constructs show efficient regulation upon metabolite recognition. Importantly, by applying riboswitch fluorescence sensing to the vast repertoire of mutant strains of the Keio collection, it basically allows to perform metabolite detection in each mutant and thus to obtain > 4000 data points per detected metabolite. Furthermore, by obtaining such "metabolic fingerprints", one may attempt to define clear expression profile per metabolite and use this information to deduce the identity of current orphan riboswitches^5,10^. Several riboswitch-based biosensors have been obtained using either native *E. coli* riboswitches or using riboswitches from other bacteria, suggesting that it should be possible to obtain a large collection of biosensors. In particular, biosensors detecting glycine^51^, purines^52^, cobalamin^13,14,53–56^, preQ1-II^57^, adenine^58^ and Ni/Co^59^ have recently been obtained. Clearly, the approach developed here could be widely applied to any current or newly-discovered riboswitches and provide invaluable tools to characterize fundamental metabolic processes^3,60^.

In conclusion, our data has revealed that riboswitches may be used as biosensors and may be used in high-throughput screen analyses with the Keio collection. Our study has successfully demonstrated that riboswitch fluorescence assays represent a powerful approach to decipher subtle metabolic perturbations caused by gene expression variations. The approach described in this study could be useful for the development of more sophisticated screen assays and for the detection of metabolites in any cellular system.

## Methods

### DNA oligonucleotides and bacterial strains

The main strains used in this study are the *E. coli* K-12 BW25113 WT and mutants from the Keio collection^11^. The plasmid constructions were done in *E. coli* DH5α, a strain known to allow a stable amplification. The conjugations were done using the *E. coli* MFD *pir* strain containing the RP4 conjugation machinery and which is auxotroph to diaminopimelic acid (DAP) to allow selection. The *pgpA* mutant remade in this study was done following the same technique used in the Keio collection^11^ only with the 5' primer shifted downstream to avoid truncating the end of the *thiL* gene.

The strains and DNA oligonucleotides used are listed in the Supplementary Tables 2 and 3, respectively.

### Plasmids construction

The plasmid backbone used comes from the Alon collection^12^ and has been modified with the Gibson assembly technique^61^ to insert genes of interest into the plasmid after linearization with the proper restriction enzymes. The resistance cassette of the plasmid has been changed from kanamycin to ampicillin to be compatible with the Keio collection which already contains one of the former. The inserts containing the desired templates (binding mutant, other riboswitches or fluorescent proteins) were produced by PCR then assembled through the same technique into the plasmid. To preserve the regulation effect, the natural *thiC* promoter was kept in every iteration, changing just the riboswitch if needed. The assembly mix was then transformed into DH5α strain and the transformants were selected on agar supplemented with ampicillin (100 µg/mL). The colonies were verified by PCR and sequencing. The plasmid was transformed in the strains of interest either by TSS transformation^62^ or by conjugation.

### Liquid fluorescent assays

The liquid fluorescent assays were performed in 96-well plates incubated for 16 h at 37 °C with double orbital agitation in a Sparks plate reader from Tecan. Precultures were grown in the media of interest (LB or M63 minimum medium) with kanamycin (50 µg/mL) or ampicillin (100 µg/mL) overnight. The solution was next diluted in fresh media at 3% in with or without supplemented TPP (0 to 1 mM) for fluorescence assays. The GFP fluorescence was monitored using an excitation of 489 nm and emission detection was performed at 535 nm. The optic density was read at 600 nm every 10 min. The blank readings were removed from the results and the Fluorescence/OD_600nm_ ratio was calculated to determine the regulation effect of TPP on the GFP expression. The effects observed for the exponential (midpoint of maximal amplitude) and stationary (16 h of growth) phases were based on readings taken at OD_600_. The condition without ligand serves as a reference for the normalization. The samples were at least tested for three biological replicates containing three technical replicates.

### Microscopy

Precultures were grown in M63 minimum medium with ampicillin (100 µg/mL) then used to inoculate fresh medium with or without supplemented 1 mM TPP and incubated at 37 °C with agitation until reaching OD_600nm_ = 0.7. The cultures were visualized on slides using a Zeiss Axio Observer Z1 epifluorescence microscope equipped with a Zeiss Axiocam 506 mono camera, a Zeiss 100X/1.4 Plan-Apochromat oil objective and GFP filter set. Zeiss Zen 2.0 software were used to capture images which were analyzed with the open-source software CellProfiler v4.2.1^63^. The images analysis, including background subtraction, cell segmentation and intensity measurements were performed. Several hundred bacteria were analyzed for each condition.

### High-throughput assays

Reporter plasmids were transformed using the TSS approach into the MFD *pir* strain and incubated O/N in LB supplemented with kanamycin (50 µg/mL) or ampicillin (100 µg/mL) and DAP (0.3 M) at 37 °C with agitation. The cultures were then transferred to a rectangular plate compatible with the Rotor HDA robot (Singer Instruments) alongside the Keio collection plated on 3 1536-kanamycin (50 µg/mL) LB plates. Conjugations were done by pinning the donor strains (MFD + reporter plasmid) on top of the Keio mutants on DAP plate for 1 h to allow the transfer of the reporter plasmid from the MFD *pir* strain to the Keio collection mutant strains. The conjugants were then pinned onto ampicillin/kanamycin LB agar plates to select for Keio mutants containing the plasmid, the MFD *pir* strain being counter-selected by the kanamycin and the absence of DAP. To ensure a proper growth and more reliable results, the plates were pinned again on new plates and pictures were taken at 4 and 24 h of growth at 37 °C using a Chemidoc (Bio-Rad) for the white light and a Typhoon FLA 9000 for the fluorescence light. Three biological replicates of the screen were performed and the data are reported in the Supplementary Data section. A Pearson analysis of three biological replicates is shown in Supplementary Table S1. The average of the replicates and the standard deviations are reported for selected candidates in Fig. 6B and G. The complete sets of data are included in a Supplementary Excel datafile.

### Colony images data analysis

The pictures were analyzed as described previously^26^ using the open-source software Fiji^64^. Briefly, the background noise was removed before delimiting regions of interest (ROIs) for each colony. The pixels integrated intensity, the sum of the pixels values not reported to their number, was extracted for the white light (growth) and the fluorescence images. The exported data are then normalized and analyzed with the open-source R software^26^. The fluorescence/biomass is calculated to mitigate the edge effect and normalize the fluorescence with the growth of each colony. The binding mutant G31C underwent the same process, and its results were used to normalize the results to remove all the variations in GFP expression not related to TPP. A threshold of 1 ± 5 standard deviation (SD) has been set in order to determine the hits. Hit lists of mutant strains crossing that threshold are generated alongside heatmaps to ensure the absence of expression patterns on the plates. The duplicated strains found on the Keio collection were analyzed separately to serve as an internal control. The graphs were generated with Python 3.11 using Pandas and Plotly for Python packages.

### Complementation assays

Some of the hits obtained were selected to undergo a complementation assay. To restore the expression of the mutated gene, a plasmid from the Aska collection was used to overexpress the otherwise deleted gene^30^. This plasmid containing a chloramphenicol resistance cassette, it was transformed into the cells containing the *thiC-gfp* plasmid using the TSS transformation technique. The transformants were selected on chloramphenicol/ampicillin LB agar plates and tested in M63 liquid culture alongside strains without the Aska plasmid to compare the recovery of the phenotype towards a potentially wilder phenotype.

### Supplementary Information

Supplementary Information 1.

Supplementary Information 2.

## Data Availability

The datasets used and/or analyzed during the current study are available from the corresponding author on reasonable request.
