# Supplementary material for: Fluorescent riboswitch-controlled biosensors for the genome scale analysis of metabolic pathways
Source: Sci Rep. 2024 May 31;14:12555. doi: 10.1038/s41598-024-61980-w (PMC11143247; doi:10.1038/s41598-024-61980-w)
Supplement: Supplementary file 1 — Supplementary Information 1. [file 41598_2024_61980_MOESM1_ESM.docx]

**Fluorescent riboswitch-controlled biosensors for the genome scale analysis of metabolic pathways.**

**A. Michaud, D. Garneau, J.-P. Côté and D.A. Lafontaine***

Department of Biology, Faculty of Science, RNA Group, Université de Sherbrooke, Sherbrooke, Quebec, Canada, J1K 2R1.

*Corresponding author. E-mail: daniel.lafontaine@usherbrooke.ca

Keywords: Riboswitch, metabolic pathway, thiamin pyrophosphate, green fluorescent protein

**
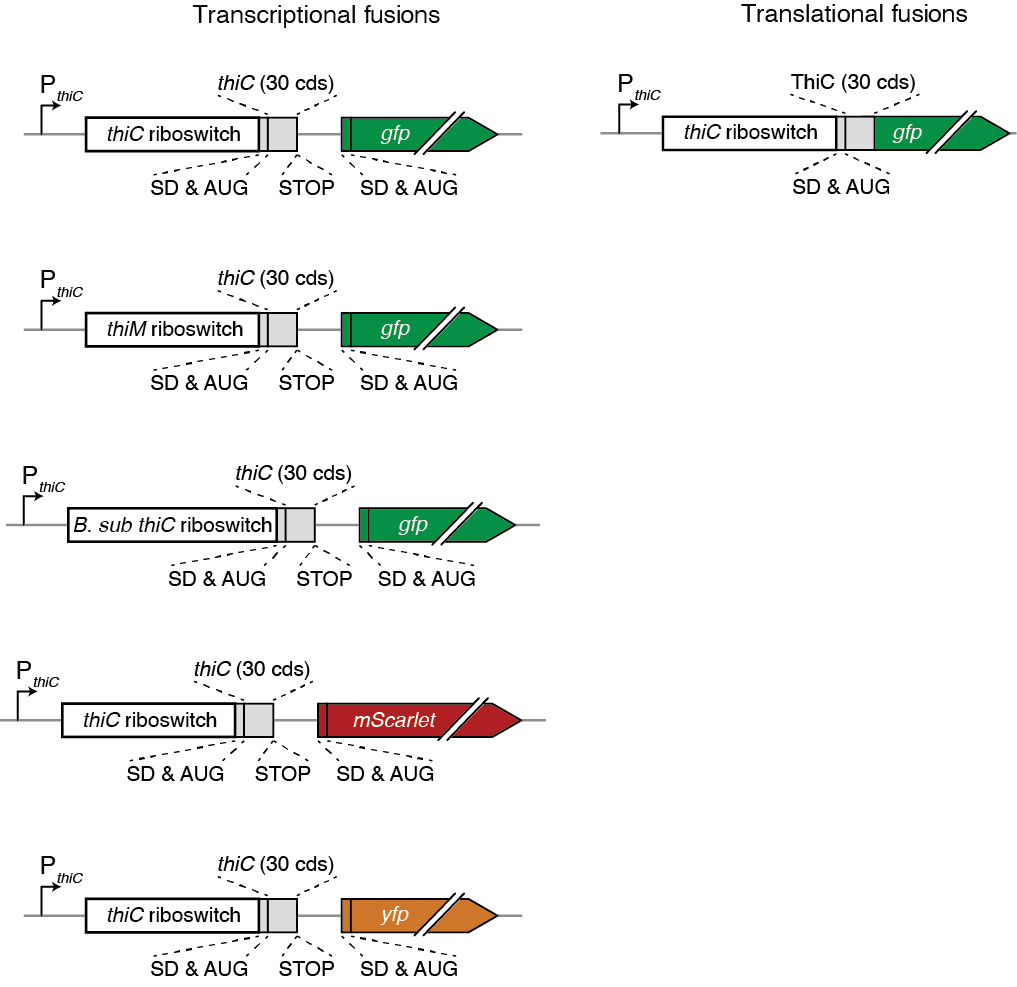
**

**Supplementary Figure 1. Schematics of constructs used in this study.**

*Left,* transcriptional constructs containing the *E. coli thiC* and *thiM* riboswitches (*thiC* and *thiM*) and the *B. subtilis thiC* riboswitch (*B. sub thiC*). Reporter genes include *gfp* (green), *mScarlet* (red) and *yfp* (dark orange). *Right*, translational construct containing the *E. coli thiC* riboswitch. SD, AUG and STOP represent the Shine-Dalgarno, start codon and stop codon sequences, respectively. Constructs are expressed from the natural *thiC* promoter (P*_thiC_*). The SD-AUG sequences of the natural *thiC* sequence corresponds to GGAAugagcuAUG and that of the *gfpmut2* to GGAGatatacatATG.

**
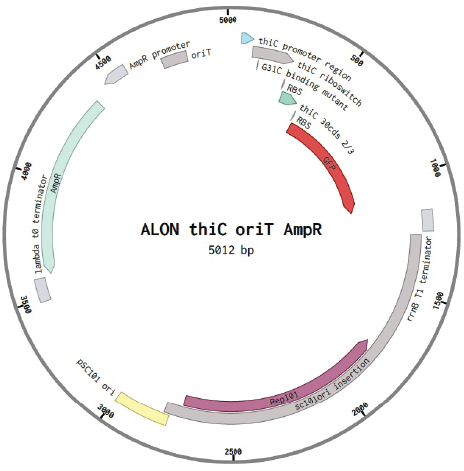
**

**Supplementary Figure 2. Plasmid map of the thiC-gfp fusion used in this study.**

The backbone of the plasmid is derived from a previous study^1^. The resistance gene has been changed to ampicillin (Amp^R^). The origin of transfer (OriT-RP4) has been derived for a previous study^2^. A variant of the construct has been made in which the G31C mutation has been introduced to prevent riboswitch regulation^3^.

**
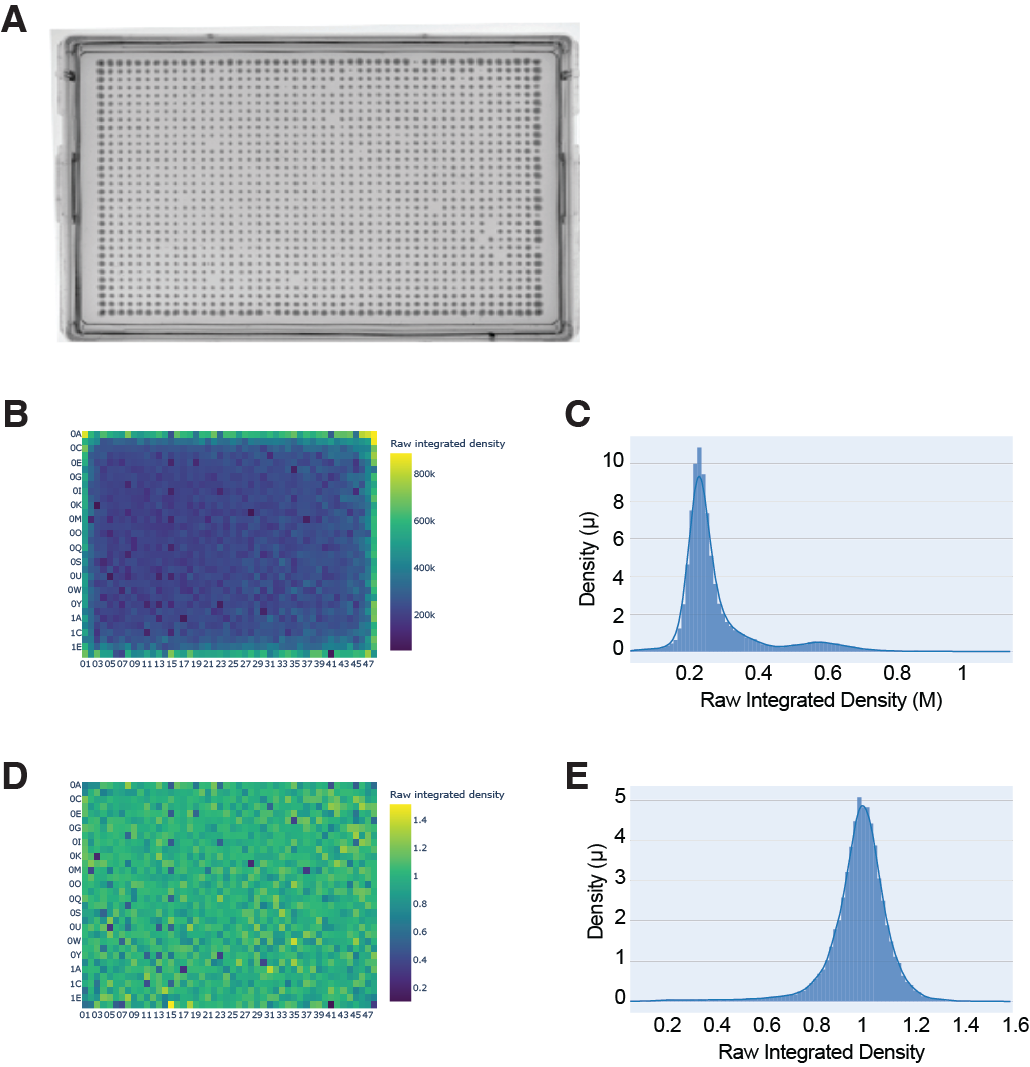
**

**Supplementary Figure 3. High-throughput array using the WT riboswitch to monitor bacterial growth.**

**(a)** Example of a high-throughput agar plate containing 1,536 strains used to monitor the growth of the Keio collection.

**(b, c)** Example of a pre-corrected heatmap of the plate representing the density of the biomass of each strain in **b**. The coordinates of the rows and columns are indicated on at the left and bottom of the image, respectively. The legend for the integrated density is indicated at the right. The quantification in **c** represented as a histogram indicates that the data are heavily skewed toward lower values and exhibit a border effect (peak at ~0.6), making difficult to compare the data across different plates^4^.

**(d, e)** Example of a post-corrected heatmap of the plate representing the biomass of each strain in **d**. The coordinates of the rows and columns are indicated on at the left and bottom of the image, respectively. The legend for the integrated density is indicated at the right. The corrected quantification in **e** indicates that the data are closer to a value of 1, which is important for plate-to-plate comparisons.

**
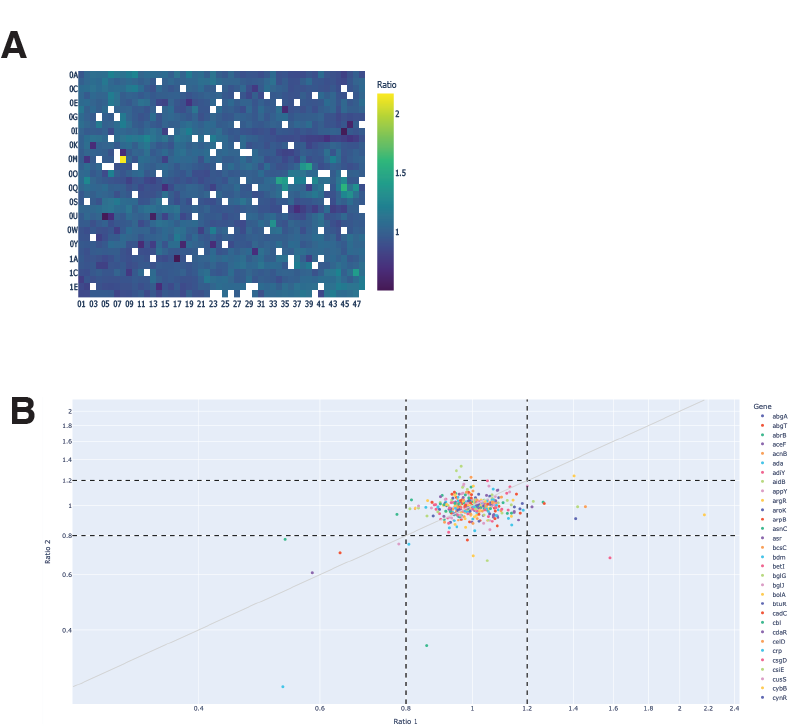
**

**Supplementary Figure 4. Heatmap representing the fluorescence/growth ratio using the WT riboswitch.**

**(a)** Example of a heatmap of the plate representing the ratio of the fluorescence/biomass of each strain. The fluorescence was obtained using the WT *thiC-gfp* transcriptional fusion. The coordinates of the rows and columns are indicated on at the left and bottom of the image, respectively. The legend for the ratio intensity is indicated at the right.

**(b)** Graph representing the ratios of duplicated strains across biological replicates the WT *thiC-gfp* transcriptional fusion. The dotted lines represent 5 standard deviation errors.

***
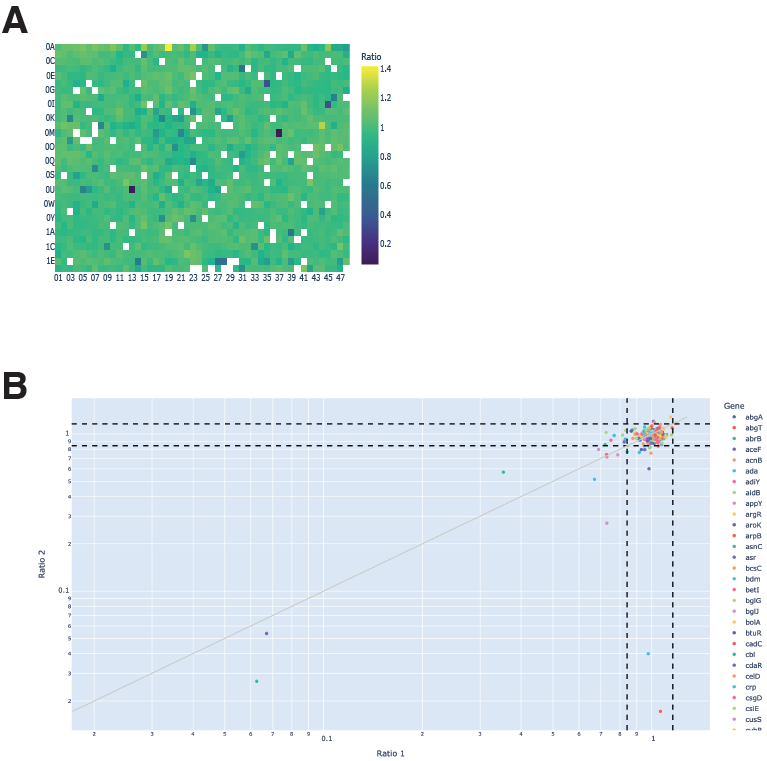
***

**Supplementary Figure 5. Heatmap representing the fluorescence/growth ratio using the G31C mutant riboswitch.**

**(a)** Example of a heatmap of the plate representing the ratio of the fluorescence/biomass of each strain. The fluorescence was obtained using the G31C *thiC-gfp* transcriptional fusion. The coordinates of the rows and columns are indicated on at the left and bottom of the image, respectively. The legend for the ratio intensity is indicated at the right.

**(b)** Graph representing the ratios of duplicated strains across biological replicates the G31C *thiC-gfp* transcriptional fusion. The dotted lines represent 5 standard deviation errors.


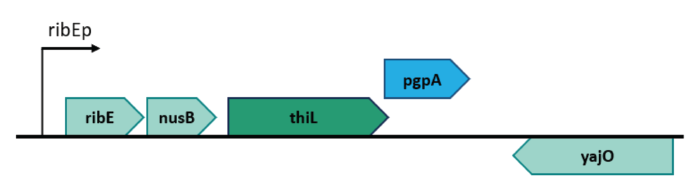


**Supplementary Figure 6. Genomic context of *pgpA*.** The gene *thiL* and *pgpA* are part of the same operon and are partially overlapping by 22 nucleotides. The operon is expressed by the ribEp promoter^5^.

**
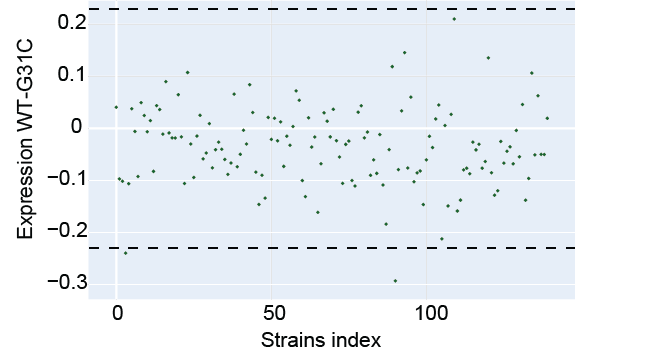
**

**Supplementary Figure 7. High-throughput analysis of TPP levels in the Storz collection.** Index plot showing the ratio *gfp* expression/strain biomass for the Storz collection. Screens were performed using the WT *thiC-gfp* transcriptional fusion and the G31C mutant of the fusion to remove any effect unrelated to riboswitch regulation. The dotted lines represent 5 standard deviation errors.

**Supplementary Table 1. Pearson analysis of the high-throughput screens using the WT and G31C riboswitch constructs.**

| **WT** | | | |
| --- | --- | --- | --- |
|  | **Replicate 1** | **Replicate 2** | **Replicate 3** |
| **Replicate 1** | 1.00 | 0.77 | 0.76 |
| **Replicate 2** |  | 1.00 | 0.83 |
| **Replicate 3** |  |  | 1.00 |
|  |  |  |  |
| **G31C** | | | |
|  | **Replicate 1** | **Replicate 2** | **Replicate 3** |
| **Replicate 1** | 1.00 | 0.76 | 0.67 |
| **Replicate 2** |  | 1.00 | 0.73 |
| **Replicate 3** |  |  | 1.00 |
|  |  |  |  |

**Supplementary Table 2. Bacterial strains used in this study.**

| **ID** | **Strain** | **Plasmid** | **Reference** |
| --- | --- | --- | --- |
| 332 AM | BW25113 | Alon *thiC-gfp,* Kan^R^ | ^1^ |
| 361 AM | MFD *pir* | *thiC-gfp*, Amp^R^, OriT | This study |
| 362 AM | BW25113 | *thiC-gfp*, Amp^R^, OriT | This study |
| 363 AM | MFD *pir* | *thiC-G31C-gfp*, Amp^R^, OriT | This study |
| 364 AM | BW25113 | *thiC-G31C-gfp*, Amp^R^, OriT | This study |
| 375 AM | BW25113 | *thiC-gfp*, TRL fusion, Amp^R^, OriT | This study |
| 381 AM | BW25113 | *thiC from B. subtilis-gfp*, Amp^R^, OriT | This study |
| 383 AM | BW25113 | *thiM-gfp*, TRX fusion, Amp^R^, OriT | This study |
| 385 AM | BW25113 | *thiC-G31C-gfp*, TRL fusion, Amp^R^, OriT | This study |
| 489 AM | BW25113 Δ*thiL* | *thiC-gfp*, Amp^R^, OriT | This study |
| 490 AM | BW25113 Δ*thiL* | *thiC-G31C-gfp*, Amp^R^, OriT | This study |
| 491 AM | BW25113 Δ*pgpA* | *thiC-gfp*, Amp^R^, OriT | This study |
| 492 AM | BW25113 Δ*pgpA* | *thiC-G31C-gfp*, Amp^R^, OriT | This study |
| 493 AM | BW25113 Δ*pgpA* (*thiL* intact) | *thiC-gfp*, Amp^R^, OriT | This study |
| 494 AM | BW25113 Δ*pgpA* (*thiL* intact) | *thiC-G31C-gfp*, Amp^R^, OriT | This study |
| 505 AM | BW25113 | *thiC-mScarlet*, Amp^R^, OriT | This study |
| 507 AM | BW25113 | *thiC-yfp*, Amp^R^, OriT | This study |

**Supplementary Table 3. Oligonucleotides used in this study.**

| **Name** | **Sequence 5’-3’** |
| --- | --- |
| FWD insert OriT-AmpR | GGCGCAGGGGATCAAGATCTGATCAAATTCGTCGCCGGCCAGCCT |
| REV insert OriT-AmpR | CTATCAACAGGAGTCCAAGCGAGCTCttaccaatgcttaatcagtgaggcacct |
| FWD G31C binding mutant | GCAGGCCAGAAGACGCGCGTTGCCC |
| REV G31C binding mutant | GGGCAACGCGCGTCTTCTGGCCTGC |
| FWD prom thiC RS thiM | caatgccccatttgcggggcctgcgatttatcatcgcaaccaaac |
| REV prom thiC RS thiM | ggttgcgatgataaatcgcaggccccgcaaatggggcattgaatg |
| FWD ALON AatII | GGCAATTCCGACGTCTAAGAAACCATTATTATCATGACATTAACCTATAAAAATAGGCG |
| FWD thiM fusion TRX GFP | cctcttgtgcactgcatgaccaatgatCTCTAGATTTAAGAAGGAGATATACATATG |
| REV thiM fusion TRX GFP | CATATGTATATCTCCTTCTTAAATCTAGAGatcattggtcatgcagtgcacaagagg |
| FWD thiC 31cds YFP | ggcaccgcctttcccaactcaaaTAAGGAGGAAAGTCACATTATGAGCAAAGG |
| REV YFP thiC 31cds | CCTTTGCTCATAATGTGACTTTCCTCCTTAtttgagttgggaaaggcggtgcc |
| FWD thiC 31cds/YFP Bba | CTCTAGATTTAAGAAGGAGATATACATatggtgagcaagggcgaggagctgttcaccg |
| REV thiC 31cds/YFP Bba | cggtgaacagctcctcgcccttgctcaccatATGTATATCTCCTTCTTAAATCTAGAG |
| REV YFP Bba ALON | CATGCCTGCAGGTCTGGACATttattacttgtacagctcgtccatgc |
| FWD thiC 31cds/mScarlet | CTCTAGATTTAAGAAGGAGATATACATatgcattcgggcgtgagcaaagg |
| REV thiC 31cds/mScarlet | cctttgctcacgcccgaatgcatATGTATATCTCCTTCTTAAATCTAGAG |
| REV mScarlet ALON | GCATGCCTGCAGGTCTGGACATttatttatacagttcgtcc |
| FWD pgpA subtstitution KanR | TTGTTTTATTCGTGACGGCGAACCTGTTACATTAGACTGGAAAGGATATGATTCCGGGGATCCGTCGACC |
| REV pgpA substitution KanR | CAAGCGTCACATCAGGCATCGGTGCACAACTACGACAGAATACCCAGCGGTGTAGGCTGGAGCTGCTTCG |
| FWD thiL substitution KanR | TTCCATGATCGCCGGCCTTTTCTTTTTTACCTGCTGAGGCATAACGTATGATTCCGGGGATCCGTCGACC |
| REV thiL substitution KanR | CGACATCTTTATGGCGTGGCAAAATGGTCATATCCTTTCCAGTCTAATGTTGTAGGCTGGAGCTGCTTCG |
| FWD deletion Keio pgpA- thiL intact | ctgaagatgagtaatccgtggcatctacttgctgtcggattcggaagtggTATGATTCCGGGGATCCGTCGACC |
| FWD seq thiL | gattcacggaagaccgttccatgatcg |
| REV seq thiL | cacttccgaatccgacagcaagtagatg |
| FWD seq pgpA | gtgacggcgaacctgttacattagactg |
| REV seq pgpA | accgcatcctgtcgtaggatttaaataagagtc |
| REV check YFP | CCATAAGTAGCATCACCCTCGCCTTC |
| REV check mScarlet | ggtttgcgtaccttcatacggacg |

**References**

1. Zaslaver, A. *et al.* A comprehensive library of fluorescent transcriptional reporters for Escherichia coli. *Nature methods* **3**, 623–8 (2006).

2. Pansegrau, W., Balzer, D., Kruft, V., Lurz, R. & Lanka, E. In vitro assembly of relaxosomes at the transfer origin of plasmid RP4. *Proc Natl Acad Sci U S A* **87**, 6555–6559 (1990).

3. Chauvier, A. *et al.* Transcriptional pausing at the translation start site operates as a critical checkpoint for riboswitch regulation. *Nature communications* **8**, 13892 (2017).

4. French, S. *et al.* A robust platform for chemical genomics in bacterial systems. *Molecular biology of the cell* **27**, 1015–25 (2016).

5. Nonaka, G., Blankschien, M., Herman, C., Gross, C. A. & Rhodius, V. A. Regulon and promoter analysis of the E. coli heat-shock factor, sigma32, reveals a multifaceted cellular response to heat stress. *Genes Dev* **20**, 1776–1789 (2006).
